# Structural, Genetic, and Functional Signatures of Disordered Neuro-Immunological Development in Autism Spectrum Disorder

**DOI:** 10.1371/journal.pone.0048835

**Published:** 2012-12-04

**Authors:** Vishal Saxena, Shweta Ramdas, Courtney Rothrock Ochoa, David Wallace, Pradeep Bhide, Isaac Kohane

**Affiliations:** 1 Department of Neurology, Massachusetts General Hospital, Charlestown, Massachusetts, United States of America; 2 Department of Medicine, Massachusetts General Hospital, Charlestown, Massachusetts, United States of America; 3 Harvard Medical School, Boston, Massachusetts, United States of America; 4 Department of Mechanical Engineering, Massachusetts Institute of Technology, Cambridge, Massachusetts, United States of America; 5 Center for Lung Biology, University of South Alabama College, Mobile, Alabama, United States of America; 6 Department of Endocrinology, Children's Hospital, Boston, Massachusetts, United States of America; 7 Department of Computational Medicine and Bioinformatics, University of Michigan, Ann Arbor, Michigan, United States of America; University of South Florida College of Medicine, United States of America

## Abstract

**Background:**

Numerous linkage studies have been performed in pedigrees of Autism Spectrum Disorders, and these studies point to diverse loci and etiologies of autism in different pedigrees. The underlying pattern may be identified by an integrative approach, especially since ASD is a complex disorder manifested through many loci.

**Method:**

Autism spectrum disorder (ASD) was studied through two different and independent genome-scale measurement modalities. We analyzed the results of copy number variation in autism and triangulated these with linkage studies.

**Results:**

Consistently across both genome-scale measurements, the same two molecular themes emerged: immune/chemokine pathways and developmental pathways.

**Conclusion:**

Linkage studies in aggregate do indeed share a thematic consistency, one which structural analyses recapitulate with high significance. These results also show for the first time that genomic profiling of pathways using a recombination distance metric can capture pathways that are consistent with those obtained from copy number variations (CNV).

## Introduction

Autism spectrum disorder, a neurodevelopmental disease with an incidence of up to 1% is increasingly recognized as a highly heterogeneous complex disorder [1], [2], [3], [4]. Genetic studies via pedigree analysis and via studying the disruptions at the nucleotide level (such as copy number variations (CNVs) or structural variations (SVs)) have been quite successful in the study of various disorders, especially in single gene or Mendelian disorders.

In Mendelian disorders, such as for example, Huntington's disease, various pedigree analyses that are conducted on different families point with remarkable consistency to the same locus. However, the results of numerous pedigree analyses in autism have mapped to different genetic loci, possibly a reflection of the non-Mendelian and complex nature of autism. Single gene approaches may fail to find underlying mechanisms in this context where an integrative approach might succeed. Moreover although there is considerable clinical heterogeneity in autism (a now prototypical spectrum disorder), there is considerable concordance ([5], [6]) amongst expert developmental specialists by the time the affected child is five years old or older. Therefore, we hypothesized that even if autism has complex etiologies, it does have an underlying molecular physiology overlap shared by autistic individuals. This overlap may occur at several levels (ranging from clinical symptoms to gene expression). Because biological pathways take direct account of mechanistic principles underlying biological function, we therefore focused on biological pathways as our level of abstraction for finding this overlap.

**Figure 1:**
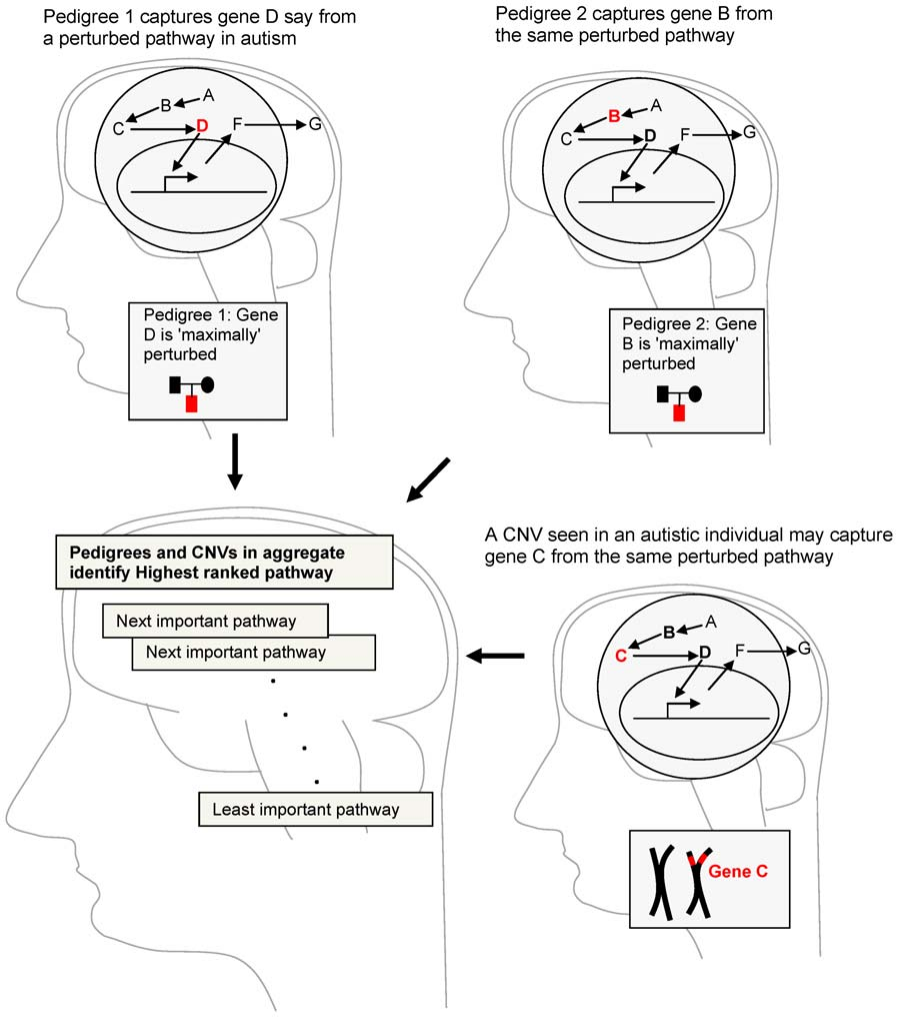
A conceptual picture of our overall analysis. Each affected individual from different pedigrees captures a different part of the same pathway. The same will be true of different CNVs in different autistic individuals.

From this perspective an affected individual from an autism pedigree (which is used to obtain linkage peaks in autism) may point to a certain gene (and thus a particular location on the genome) within a common pathway perturbed in autism. Another pedigree may point to a different location within the same pathway. The same may be true of structural perturbations in the genome (Copy Number Variations (CNVs) or Structural Variations) with each affected individual's CNVs capturing different aspects of the same common pathway. Figure 1 illustrates this concept and the idea is captured in a methodology called Linkage ordered Gene Sets (LoGS) that we present in this paper.

**Figure 2:**
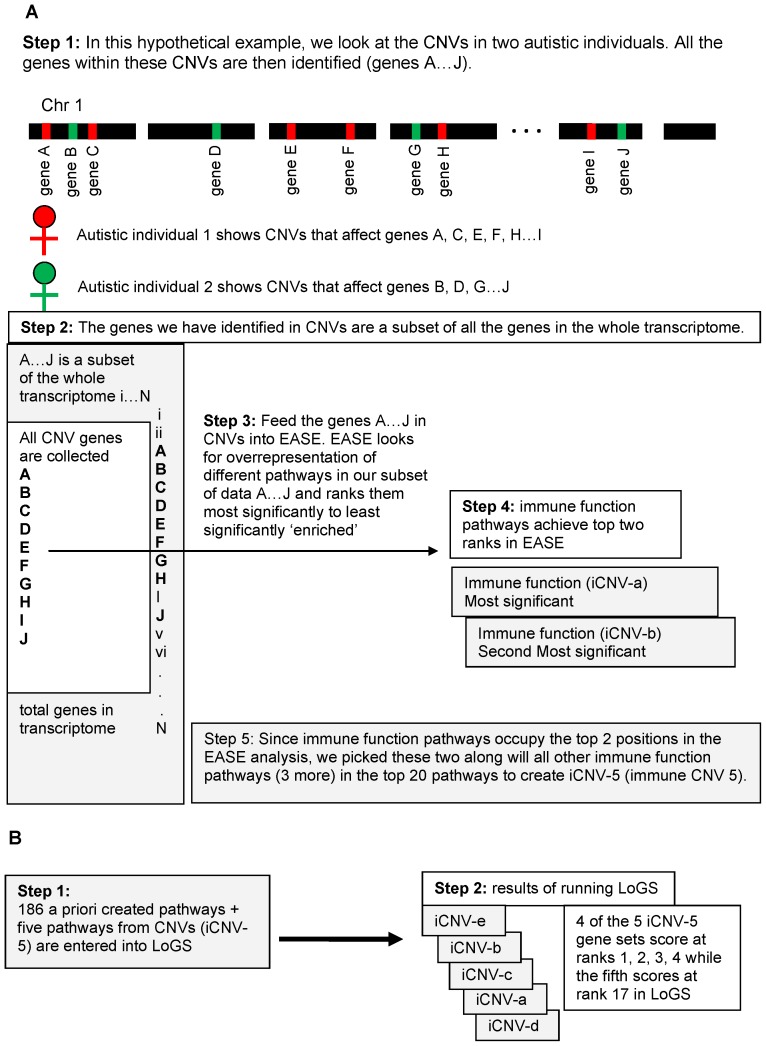
Overall analysis scheme. All genes within CNVs were used to find the top ranked pathways in the CNVs (A) and these new pathways along with other a priori created pathways were tested using LoGS (B).

LoGS takes pre-existing gene sets and ranks them in terms of their importance in autism. To integrate CNV studies with LoGS, we first looked for pathways that were perturbed in CNVs of autistic individuals (Table S1). The top two ranked pathways from the CNV analysis were both immune function related. With these top ranked pathways we identified three other immune related pathways located in the top 20 sets from the CNV analysis and aggregated these into 5 new gene sets (individually referred to as iCNV-a through e and collectively as iCNV-5 for immune CNV 5 sets). These iCNV-5 gene sets along with 186 other *a priori* created gene sets were then tested in LoGS as summarized in Figure 2.

**Figure 3:**
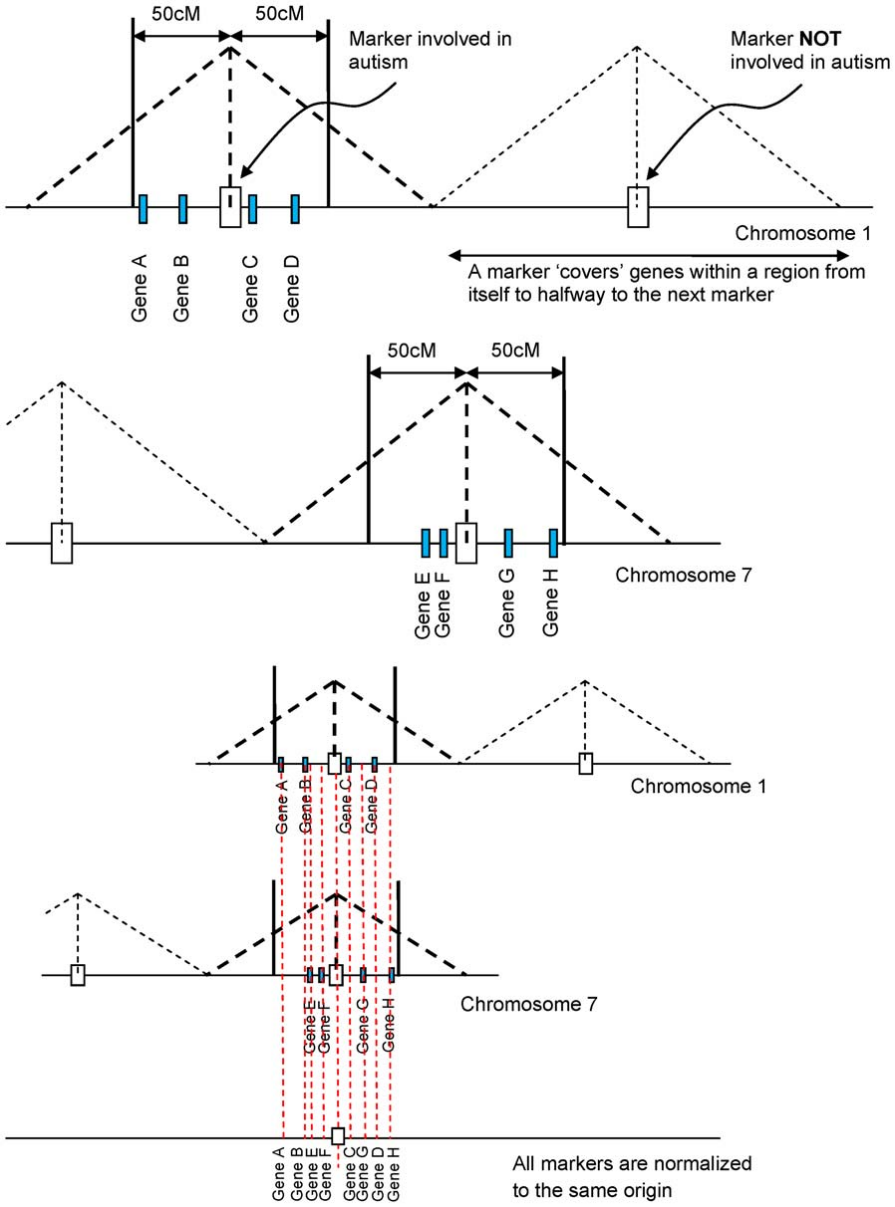
General overview of LoGS. We pick markers on various chromosome implicated in autism. We then find genes within 50 cM of each marker. Next we ‘align’ each marker to have the same or common origin and then rank genes from this common origin.

**Figure 4:**
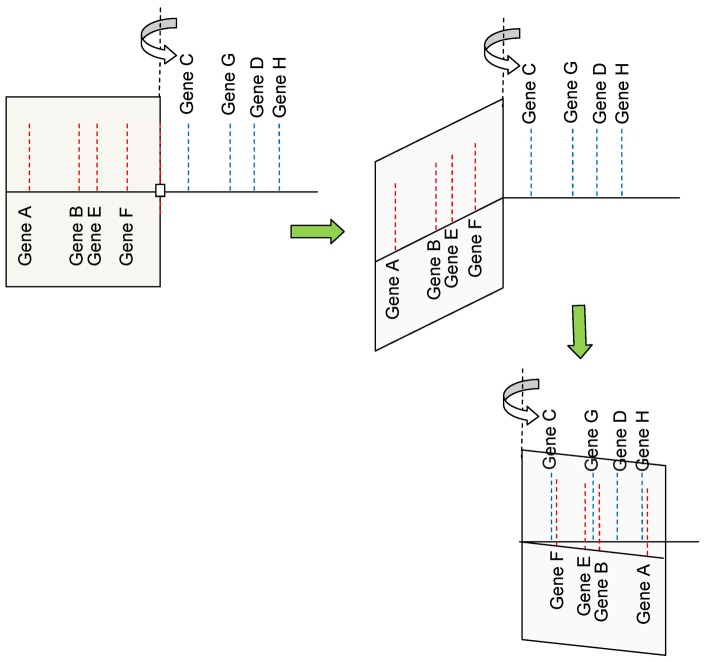
Genes to the left and right of the marker are treated equally (LoGS overview continued). Here we show how left ranked genes and right ranked genes are placed together in the same ranking.

LoGS is based on the idea that various loci obtained from pedigree studies can be used to rank previously compiled pathways important in that disease. This ranking is obtained through a ranking of all genes linked to that locus using genetic distance (within different sized linkage windows). Consider two markers that have been identified in two pedigree studies, one on chromosome 1 and another located on chromosome 7. We first find all genes within a 50 cM window on either side of the marker on chromosome 1 and repeat again for the marker on chromosome 7. We then combine the markers such that they both sit at the origin (see Figure 3) and then rank all genes within 50 cM of these two markers in terms of their distance from this combined origin. Since distances to the left of the combined origin are equivalent to distances to the right (we are only interested in the distances of the genes from this common origin), we rotate the left hand genes from the origin into the right hand side (or take the absolute value). This is shown in Figure 4 (see methods section for details). 50 cM windows on either side of the loci are chosen because that is the limit of linkage and because choosing this window size allows the largest number of genes that may be responsible.

**Figure 5:**
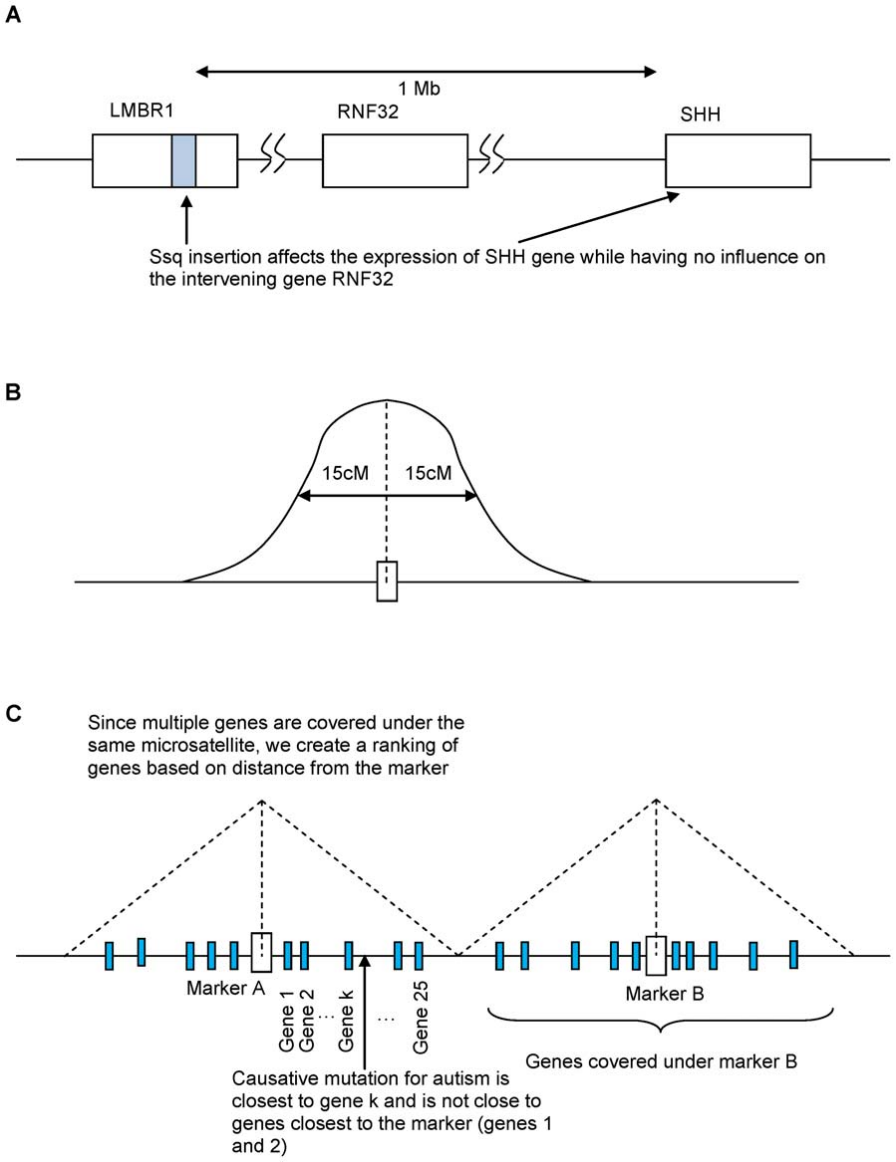
Why the closest gene is not necessarily the best gene. A. Far away genes can be influenced by genes closer to a marker. Thus, we can't just use the closest genes to the marker. B. Since our real locus could be anywhere within the 30 cM window, any of the genes within the window could be the closest gene, and since our best location for the marker is the center of the window, we simply rank the genes from this point to take into account the fact that any of the genes within the window could be the gene closest to some ‘real’ marker. C. The low density of markers means that many genes are ‘covered’ by each marker. The gene of interest may be far from the marker and may not necessarily be the closest gene from the marker.

Researchers typically take a marker and use the closest gene to that marker as an important gene in that disease. Our rationale for using all genes within a certain sized window rather than the closest flanking genes is based upon the following ideas:

1. Both flanking and some non flanking genes next to the locus may be important.
2. The locus itself may be important. However, its importance may influence genes that are not the closest to it. A disruption can occur in the genome that may influence non flanking genes [7], [8], [9]. For example, according to Kleinjan “A complex hotspot for limb abnormalities is found 1 Mb upstream of SHH, within the introns of LMBR1. The region contains a conserved noncoding element that is capable of functioning as an enhancer that drives SHH expression in the limb bud in both an anterior and posterior zone, as well as a repressor element that silences the anterior expression. The Sasquatch insertion disrupts the anterior repression function, whereas the acheiropodia deletion is thought to disrupt positive enhancer activity.” Another gene RNF32 sits between the region of control and the SHH gene that is controlled [7]. Further, Introns can contain microsatellites [10], [11]. Please see Figure 5A (this figure is adapted from [7]).
3. Even if the closest gene(s) to the locus is (are) the most important, we just don't have the exact location of the locus. There may be uncertainty of 15 cM on either side of the locus [12]. As shown in Figure 5B in the absence of further information, we place the measured marker at the center of the region of uncertainty and therefore consider genes not merely adjacent.
4. Microsatellite marker density in pedigree analysis is low and consequently the signal for the correct location affecting the disease may arise at a distance from the marker. For example, Yonan *et al.*'s study [13] of autism susceptibility loci used 408 markers. Since a conservative estimate for the number of genes on the human genome is approximately 20,000 genes, (which on average are 10–15 Kb [14]), and thus microsatellite markers on average are spaced 50 genes apart. Therefore the closest gene to the marker may not be the gene with the etiological variant (Figure 5C). It is worth noting that similar considerations at a different scale occur with much higher density markers more typical of GWAS [15].

Using LoGS, we show that integrated results of linkage studies are highly congruent with those obtained from copy number variation profiles of individuals with autism as compared with those of controls. This congruence points to a common set of pathways previously implicated in immunological response, inflammation, and development. Moreover, the top 2 ranked gene sets in CNVs ranked within the top 4 LoGS sets, and the iCNV-5 gene sets claimed the top 4 ranks (as well as rank 17) in LoGS.

## Results

### Structural variations in autism

The group of genes that reside wholly within structural variation regions were found to be enriched (using the EASE [16] Gene Ontology enrichment program) for 5 sets implicated in immune processes (Table S2). The results of EASE enrichment over CNV genes are shown in Table S1 where we present the 20 top enriched categories. These gene sets obtained ranks 1, 2, 10, 17, and 19), and are heretofore referred to as iCNV-5 (iCNV-a through e)—the Bonferroni corrected p-values for the 2 top ranked sets (both pertaining to immune function) are 2×10−4 and 1.7×10−4.

To gain further insights into the CNV based immune function gene sets that were generated, we took all the genes within the iCNV-5 gene sets and reviewed the primary CNV data to see if there was copy number gain or loss (Table 1). All the chemokines show a copy number gain while all the interferons show a loss. Interestingly, ethanol oxidation, ethanol metabolism, and alcohol dehydrogenase activity feature prominently in the CNV analysis (scoring at ranks 4, 5 and 14 respectively).

**Table 1:** Copy number gain or loss in the iCNV-5 genes.

Gene symbol	Gain/Loss
CCL1	Gain
CCL11	Gain
CCL13	Gain
CCL2	Gain
CCL7	Gain
CCL8	Gain
BMP15	Gain
FAM3C	Loss
RNF4	Gain
IFNA10	Loss
IFNA14	Loss
IFNA2	Loss
IFNA21	Loss
IFNA4	Loss
IFNA5	Loss
IFNA6	Loss
IFNA8	Loss
IFNA17	Loss
IFNB1	Loss
IFNW1	Loss
IL11	Loss
TNFSF15	Loss
MX1	Loss
MX2	Loss

### Gene sets relating to immune function and development score highly in LoGS

When LoGS was run over a set of linkage studies (for 6905 genes within 50 cM of at least one of the linkage peaks) we found that iCNV-5 was highly ranked by LoGS (Table 2). When we restricted LoGS to only those genes that were in CNV's and that were within 50% recombination distance of the linkage peaks, we were left with 319 genes. LoGS ranked the gene sets as shown in Table S3. The five immunological and inflammation gene sets (iCNV-5) again ranked topmost. Following the iCNV-5 immune gene sets, pathways enriched for development (c6 gene set) and neurogenesis (c34 gene set) score highly. Pathways labeled, by the GSEA developers [17], as c6 and c34 were the fifth- and sixth- most highly ranked, respectively. The gene memberships from these two sets were then analyzed with EASE [18] to find Gene Ontology categories enriched in each of these gene sets. The c6 gene set was enriched for epidermal differentiation and ectoderm development and the c34 gene set was overrepresented with genes annotated as involved in ‘neurogenesis’ and ‘hydrolase activity, acting on acid anhydrides’. Tables S4 and S5 list the gene memberships of these two gene sets and their top enriched categories as determined by EASE.

**Table 2:** LoGS on autism loci. Shown are the top 20 pathways.

	Gene set	V	P
1	Cytokine activity (**iCNV-e**)	255	0.005
2	Hematopoietin/IFN-class cytokine receptor binding (**iCNV-b**)	212	0.007
3	Response to virus (**iCNV-c**)	174	0.003
4	IFN-α/β receptor binding (**iCNV-a**)	173	0.002
5	c6: epidermal differentiation (BP), ectoderm development (BP)	168	0.009
6	c34:hydrolase activity (MF), neurogenesis (BP)	126	0.016
7	MAP00960_Alkaloid_biosynthesis_II	119	0
8	OXPHOS_HG-U133A_probes	119	0.01
9	c1:cellular process (BP), cell proliferation (BP)	118	0.011
10	c10:glutathione transferase activity (MF), epidermal differentiation (BP)	116	0.007
11	MAP00531_Glycosaminoglycan_degradation	108	0.007
12	c33 (proteasome complex (CC), synaptic transmission (BP))	105	0.011
13	MAP00680_Methane_metabolism	103	0.006
14	c28:signal transducer activity (MF), lactose metabolism (BP)	102	0.011
15	MAP00193_ATP_synthesis	101	0.003
16	MAP03070_Type_III_secretion_system	101	0
17	Antiviral response protein activity (**iCNV-d**)	100	0.005
18	c31:transcription factor activity (MF), cell communication (BP)	100	0.012
19	c3:ribonucleoprotein complex (CC), apoptosis (BP)	99	0.011
20	MAP00190_Oxidative_phosphorylation	97	0.005

Gene sets that begin with ‘c’ are further tested in EASE for their top categories. BP = biological process; CC = cellular component; MF = molecular function. V = enrichment score for a pathway. P = P value via permutation test.

### Significance Determination

To determine the statistical significance of the results of the LoGS analysis, a permutation test was adopted. The ranks of the genes that are within the 50 cM recombination distance of the linkage peaks that were used in our analysis were permuted and then tested for the top ranked gene sets in 1000 runs. The *p* value was then computed as the number of times a particular gene set obtained top rank in the 1000 runs divided by 1000. We note that all the gene sets tested show significance at the 0.05 level (Table 2).

### Effect of linkage window size

Because 50 cM is a relatively large distance over which to study the effects of linkage from a locus, we took different distances from the loci to see how sensitive our results are to the size of our window. We tested five smaller windows: 40 cM, 30 cM, 20 cM, 10 cM, and 5 cM. These results are presented in Table S6. We note that shrinking the distance around the loci down to 5 cM from 50 cM substantially preserves the results.

### LoGS without LOD score normalization

Next we tested how sensitive our analysis is to the LOD score normalization that is used as one step in our LoGS analysis by removing this normalization. Our strategy for the LoGS analysis started by taking a cutoff threshold of 3 for the LOD score for any linkage peak to be part of our analysis. Since this is a highly significant LOD score, relatively few studies were expected to surpass this LOD score substantially. Further, most of the LOD scores of studies that were above 3 were close to this number. Thus, we expected our results to remain substantially the same when the LOD score normalization was removed from our study. The results of running the LoGS without the LOD score normalization are presented in Table S7, and we see that in fact our results remain essentially the same.

## Discussion

With two different genome analyses, LoGS and CNV, immune system and developmental pathways appear to be involved in autism. These data are remarkably consistent. The linkage loci used in LoGS were compiled from diverse sources spanning over a decade. The CNV studies were performed recently by a different set of investigators with a study population of minimal overlap with the subjects in the linkage studies. In LoGS, the top ranked gene sets (iCNV-5) were those obtained from the CNV analyses. After iCNV-5, the next highest gene sets related to development (organogenesis and neurogenesis). Further, not only were 4 of the new gene sets (iCNV-5) at the very top of the LoGS analysis, but the developmental theme obtained using LoGS was recapitulated in the CNV analysis with developmental themes at ranks 3, 6, 12, 13, and 18 in the top 20 over-represented pathways. In toto these results coherently point to functional and genomic differences in autism related to immune function as well as development.

Prior work as reviewed in [19] has implicated immune function in autism histologically in brain and blood, in the expression of proteins in brain and blood, and in several epidemiological studies. Vargas et al. used immunohistochemistry, cytokine protein arrays, as well as enzyme linked immunosorbent assays in postmortem brain samples of autistic individuals and found significant activation of microglia, astroglia, and neuroglia in the cerebral cortex, white matter as well as the cerebellum [20]. Others have expression profiled with DNA microarrays using post mortem brain tissue from autistics reporting that “Overall, these expression patterns appear to be more associated with the late recovery phase of autoimmune brain disorders, than with the innate immune response characteristic of neurodegenerative diseases” [21]. Peripheral blood transcriptional profiling in autistic children (not suffering from other disorders such as fragile X mental retardation) showed increased expression of Natural killer cell receptors and effector molecules [22]. Proteomic profiling of blood serum from autistic children showed an increase in complement proteins [23].

In utero infections have been reported to predispose the growing fetus to developing autism and schizophrenia [24] with infections during the earlier parts of pregnancy showing progressively more severe phenotypes. Respiratory infections as well as infections with Rubella during pregnancy may predispose the growing fetus to developing schizophrenia [25]. Mouse models of autism further strongly suggest a role for the immune system in autism. Ponzio et al showed “immune mechanisms, in general, and cytokine dysregulation, in particular, as contributing factors in their [autism spectrum disorder] etiology” [26]. It is also becoming increasingly clear that the same ligands and receptors employed by the immune system play a role in the development of the central nervous system and in its functioning in the mature brain [27], [28], [29]. This raises the question of whether the immune/inflammatory signature we have found across the two genomic measurement modalities in ASD is part and parcel of the developmental disorder, a consequence of that disorder, or its trigger.

Nonetheless, it is striking that of the genes implicated by LoGS, there is a loss of genomic copies in the interferon alphas (IFNA10, IFNA14, IFNA2, IFNA21, IFNA4, IFNA5, IFNA6, IFNA8, IFNA17) and gain of copies in the “C-C” motif chemokine ligands (CCL1, CCL11, CCL13, CCL2, CCL7, CCL8) as summarized in Table 1. Several of these chemokines have been found to be overexpressed in inflammatory diseases such as ulcerative colitis [30], atopic dermatitis [31], rheumatoid arthritis [32], and in neurocognitive disorders [33]. This suggests an etiological basis for the disordered innate immunity response found in autism [34], particularly as mediated by monocytes [35] and the histologically related microglia [20], [36], [37]. The loss of interferon alpha copies, usually implicated in the response to viral infection and another component of innate immunity, could also account for a dysregulated, secondary or compensatory response of interferons and chemokines. Several of these messengers “…are produced by neurons and glia in the adult brain, and that they can acutely influence synaptic transmission.” [38]. Certain neurotrophins (which are also released by immune cells [39] cause activity-dependent changes in neural circuits in development [38].

The above could be suggestive of a link between in utero infections and brain development in the child. Thus, the genetic background by itself would not be enough via this view to cause a deranged developmental process which would rather only occur in the presence of relevant infections. Interferons are important in the control of viral infections via the induced expression of interferon-stimulated genes [40]. The loss of copy number in the interferon genes suggests a possible reduced expression of such genes when stimulated. Thus, a viral infection would last longer under such a genetic background. Viral infections also lead to the expression of various chemokines in the CNS [41]. Further, chemokines are also involved in brain development [27], [41]. There would therefore be a longer generation of chemokines and other cytokines that could interfere with normal brain development. Further, gain in copy number in chemokines may lead to higher levels of these chemokines and would thus exacerbate the derangement in brain development.

LoGS is agnostic to the type of marker used in the analysis (microsatellites, SNPs etc). SNPs could be exclusively used from GWAS studies [42]. However, the success of this method will be highly dependent on the nature of the original studies. Given that the majority of the more recent SNP population investigations are association rather than linkage studies, the efficacy of LoGS in these settings will depend on the distributional and “penetrance” characteristics of the genomic variants across the spectrum of autism. These characteristics remain to be determined. Others have tried to intersect data and findings. For example, Raychaudhuri describes a method to find the most important locus or gene from various loci obtained in a disease [43] while Hannum looks for clusters of genes relevant in a disease [44]. While these are both interesting studies, neither looks for functionally important pathways that could be relevant to a disease as does LoGS.

The results presented in this paper show that immune function may play a critical role in the genesis, development, or manifestation of autism.

## Methods

### Linkage Ordered Gene Sets

In linkage studies, the closer a gene is to a locus associated with a disorder the more likely it is to be involved in the disorder. The commonly used genetic distance measures the distance as a function of recombination events. In LoGS, all the linked genes (<50% recombination) on the chromosome with the marker are ranked as a linear function of genetic distance from the marker. However, each marker has a particular probabilistic relationship with the trait/disease being studied often quantified by a LOD score. We therefore adjust the rankings of each gene with respect to a marker by dividing the genetic distance by the LOD score. We then test a large number of a priori generated gene sets using this ranking metric to test for non-random distribution of these gene sets across the ranked list of genes in the manner of Gene Set Enrichment Analysis [17].

Twenty nine genetic loci implicated in autism in the research literature with each locus having an LOD score greater than 3 were chosen to be the inputs in the LoGS (Table S8). When there was more than one LOD score reported in the literature for a locus (entries 20, 21), the lower score was used to be conservative with respect to that locus.

By using recombination rates pertaining to each known SNP location from the Hapmap.org website in combination with the location of all genes from the ensemble.org website, we were able to determine the genetic distance of all genes within each of the chromosomes in Table S8. We searched for SNPs within each gene. When a gene had more than one SNP, we obtained the genetic distances of the SNPs towards the two ends of the gene and these averaged gave us the genetic distance for that gene (when a gene contained only one SNP, the genetic distance for that SNP served as the distance for the gene). Genes without SNPs weren't used.

**Figure 6:**
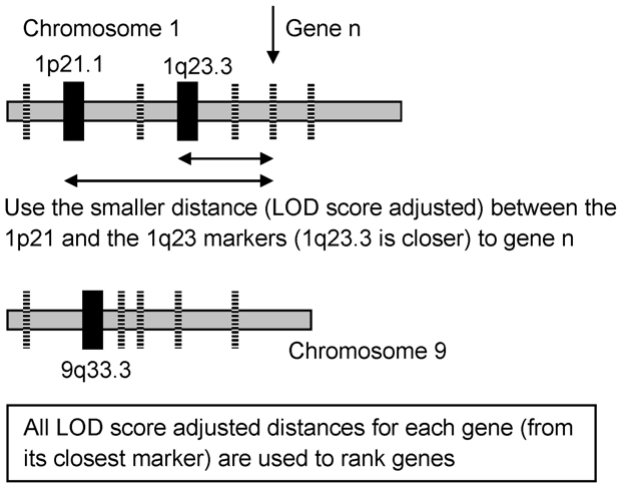
The rationale behind LoGS. In this figure, we use two loci to illustrate how LoGS works. Say Chromosome 21 has two loci that were implicated in ASD while chromosome 9 has just one locus. We then locate all the genes on chromosomes 1 and 9 and then rank them by their genetic distance from the closest locus on that chromosome (for example the gene between loci 1p21.1 and 1q23.3 is closer to 1q23.3 and thus its distance from 1q23.3 is used). This ranking for all chromosomes (in this example chromosomes 1 and 9) is then collected and we run gene set enrichment analysis as explained in the methods section. The black boxes are markers and the dashed lines represent genes.

To find the location of the autism markers, we obtained the average location in base units from the range in base units for each marker. We then found the SNP closest to this average range, and the genetic distance in recombination units pertaining to that SNP was then assigned to that marker. Next, each of these distances was then translated such that the origin was placed at the location of the marker. This new coordinate system then had genes either at negative or positive locations vis-à-vis the particular marker. The absolute value of each gene's location was taken and if there were two or more markers or loci on the same chromosome, we took the smallest of all the distances of each gene to all the loci (after we had adjusted for the LOD scores). Further, only genes within 50% recombination units of any maker were chosen in the study. The location of each gene was then divided by the LOD score for the marker used for referencing that gene. All genes from all chromosomes implicated in the linkage studies were then ranked using this final metric. This ranked system was then used to obtain the enrichment score, V, for each gene set tested as outlined previously [45], [17]. Figure 6 shows this approach for two chromosomes with 3 markers or loci.

We found the exact location of each of these loci associated with the disease from the literature. Once we obtained a ranking of all (linked) genes to all markers, we then took pre-existing gene sets (which were appropriately filtered to only have the subset of genes from each gene set that is linked to the markers) and calculated the ‘enrichment’ score for each gene set along the same lines outlined previously [45], [17].

*A priori* gene sets were created as previously reported [46]. The ranked genes over which we tested these gene sets were the subset (numbering 6905) of all genes in the genome that fall within 50% recombination distance of the linkage peak.

### Structural variations analysis

We used genome-wide structural variation studies for independent selection of common ASD pathways.

Marshall et al [47] have studied the occurrence of structural variations in autism spectrum disorder. They used 500k SNP chips to obtain regions showing these variations (described at http://projects.tcag.ca/autism_500k/). We found all genes that completely resided within each of these structural variation regions, and then used EASE [18] to define the molecular themes across these CNVs. This EASE analysis identified **immune related and developmental pathways** (Table S1).

## Supporting Information

Table S1 CNV genes in EASE. The top 20 categories in EASE are shown along with the genes (represented by gene symbols) in those categories. Shown in order are: gene set; EASE score P values adjusted for multiple testing; gene symbols.
(DOCX)

Table S2 Immune function gene sets from the copy number variation (CNV) regions of autistic individuals.
(DOCX)

Table S3 Results of LoGS analysis using only genes that were both within 50% recombination distance of the autism loci AND overlapped with CNV's. V = enrichment score.
(DOC)

Table S4 Genes in the c6 and c34 gene sets under the LoGS analysis.
(DOC)

Table S5 Top Enrichment themes of the c6 and c34 gene sets using EASE.
(DOCX)

Table S6 LoGS was rerun with different sized windows (forty percent, thirty percent, twenty percent, ten percent, and 5 percent recombination units). V = enrichment score.
(DOCX)

Table S7 LoGS without LOD: Except for two gene sets in the lower part of the top 20 ranking all the other gene sets are consistent across the LoGS which use the LOD score and the LoGS without the use of the LOD score. V = enrichment score.
(DOCX)

Table S8 LoGS data input.
(DOCX)
