# Supplementary material for: Structural, Genetic, and Functional Signatures of Disordered Neuro-Immunological Development in Autism Spectrum Disorder
Source: PLoS One. 2012 Dec 4;7(12):e48835. doi: 10.1371/journal.pone.0048835 (PMC3514226; doi:10.1371/journal.pone.0048835)
Supplement: Table S1 — CNV genes in EASE. The top 20 categories in EASE are shown along with the genes (represented by gene symbols) in those categories. Shown in order are: gene set; EASE score P values adjusted for multiple testing; gene symbols. (DOCX) [file pone.0048835.s001.docx]

**Table S1.** CNV genes in EASE. The top 20 categories in EASE are shown along with the genes (represented by gene symbols) in those categories. Shown in order are: gene set; EASE score P values adjusted for multiple testing; gene symbols.

| **CNV pathways** |
| --- |
| **1) Interferon-alpha/beta receptor binding; Molecular function; 9.10^-8^**  IFNA10; IFNA13; IFNA16; IFNA17; IFNA2; IFNA21; IFNA4; IFNB1; IFNW1 |
| **2) Hematopoietin/interferon-class (D200-domain) cytokine receptor binding; Mol Func; 7.10^-6^**  IFNA10; IFNA13; IFNA14; IFNA16; IFNA17; IFNA2; IFNA21; IFNA4; IFNA5; IFNA6; IFNA8; IFNB1; IFNW1; IL11 |
| **3) Organogenesis; Biological Process; 10^-5^**  AHSG; ANGPT2; ANKH; ANKRD7; APBA2; ARSE; ATOH1; BMP3; BMPR1B; BTD; C4ORF6; CCL2; CD164; CHRNA4; CHST8; CLN8; COL10A1; COL6A3; CRMP1; CRYAA; CYP46A1; DGCR14; DGCR2; DGCR6; DMP1; DMRT1; DMRT2; DOC2A; DRD1; DSCAM; DSP; DSPP; EBP; ELN; ETS2; EVC; EVL; FABP7; FGF12; FGF5; FGFR3; FOXC2; FOXF1; FOXP2; FZD9; GBX2; GDF10; HAND2; HDAC4; HEY2; IBSP; IFRD1; IL11; IRX4; KCNQ2; LAMA2; LIMK1; MAPK12; MBP; MEPE; MSI1; MSX1; MT3; MYH13; MYH8; MYL5; MYOM2; NAGLU; NCOA4; NDN; NR2E1; NR4A2; PAX4; PCP4; PHYH; PITX1; PKD2; PMP22; PPP2CA; PTPRZ1; RET; RFNG; RTN4R; SEMA3A; SEMA3D; SEMA5A; SEMA7A; SGCG; SHOX; SHOX2; SLIT2; SNCA; SPON2; SPP1; STMN3; T; TBX1; TBX6; TCF15; TNRC4; TUFT1; UBE3A; UFD1L; UGCG; UNC5C; WAS; WFS1 |
| **4) Ethanol oxidation; Biological Process; 10^-5^**  ADH1A; ADH1C; ADH4; ADH5; ADH6; ADH7 |
| **5) Ethanol metabolism; Biological Process; 4.10^-5^**  ADH1A; ADH1C; ADH4; ADH5; ADH6; ADH7 |
| **6) Morphogenesis; Biological Process; 7.10^-5^**  AHSG; ANGPT2; ANKH; ANKRD7; APBA2; ARSE; ATOH1; ATP10A; BMP3; BMPR1B; BTD; C4ORF6; CCL2; CD164; CHRNA4; CHST8; CLN8; CLTCL1; COL10A1; COL6A3; CRMP1; CRYAA; CYP46A1; DGCR14; DGCR2; DGCR6; DMP1; DMRT1; DMRT2; DOC2A; DRD1; DSCAM; DSP; DSPP; EBP; ELN; ETS2; EVC; EVL; FABP7; FAT; FGF12; FGF5; FGFR3; FOXC2; FOXF1; FOXP2; FSCN2; FZD9; GBX2; GDF10; HAND2; HDAC4; HEY2; HIRA; IBSP; IFRD1; IL11; IRX4; KCNQ2; LAMA2; LIMK1; MAPK12; MBP; MEPE; MSI1; MSX1; MT3; MYH13; MYH8; MYL5; MYOM2; NAGLU; NCOA4; NDN; NR2E1; NR4A2; PAX4; PCP4; PHYH; PITX1; PKD2; PMP22; PPP2CA; PTPRZ1; RET; RFNG; ROD1; RTN4R; SEMA3A; SEMA3D; SEMA5A; SEMA7A; SGCG; SHOX; SHOX2; SLIT2; SNCA; SPON2; SPP1; STMN3; T; TBX1; TBX6; TCF15; TNRC4; TUFT1; UBE3A; UFD1L; UGCG; UNC5C; WAS; WFS1; WHSC1 |
| **7) Cell-cell signaling; Biological Process; 10^-4^**  ADAM10; ADM; ADRA2C; AKAP5; AVP; BMP3; CACNA1F; CCL1; CCL11; CCL13; CCL2; CCL7; CCL8; CHAT; CHRNA4; CHRNA7; CHST4; COLQ; COMT; CPLX1; DLGAP2; DOC2A; DRD1; EDN2; ESR2; FAM3B; FAT; FGF12; FGF5; GABRA5; GABRB3; GABRG3; GALR1; GRID2; GRM1; GRM8; HTR1B; HTR7; IFNA10; IFNA17; IFNA2; IFNA21; IFNA4; IL11; INSL4; KCNIP4; KCNQ2; LEP; MAPK1; MBP; MERTK; NEO1; NMB; NOS1; OPRK1; PDGFA; PMP22; QPRT; SEMA5A; SPP1; SRD5A1; SST; TNFRSF11A; WISP3; WNT16; WNT2 |
| **8) Copper ion homeostasis; Biological Process; 2.10^-4^**  MT1A; MT1B; MT1F; MT1G; MT1H; MT1X; MT2A |
| **9) Alcohol dehydrogenase activity\, zinc-dependent; ; Molecular function; 3.10^-4^**  ADH1A; ADH1C; ADH4; ADH5; ADH6; ADH7; FASN; RTN4IP1 |
| **10) Response to virus; Biological Process; 4.10^-4^**  CCL11; CCL8; IFNA10; IFNA17; IFNA21; IFNA4; IFNA6; IFNB1; IFNW1; TLR3 |
| **11) Heavy metal sensitivity/resistance; Biological Process; 6.10^-4^**  MT1A; MT1B; MT1F; MT1G; MT1H; MT2A |
| **12) Neurogenesis; Biological Process; 8.10^-4^**  APBA2; ATOH1; BTD; C4ORF6; CHRNA4; CHST8; CLN8; CRMP1; CRYAA; CYP46A1; DGCR14; DOC2A; DRD1; DSCAM; EVL; FABP7; FGF12; FGF5; FOXP2; FZD9; GBX2; HDAC4; HEY2; IFRD1; KCNQ2; LIMK1; MBP; MSI1; MT3; NAGLU; NDN; NR2E1; NR4A2; PCP4; PHYH; PMP22; PTPRZ1; RFNG; RTN4R; SEMA3A; SEMA3D; SEMA5A; SEMA7A; SHOX2; SLIT2; SNCA; SPON2; STMN3; TNRC4; UBE3A; UNC5C; WFS1 |
| **13) Skeletal development; Biological Process; 9.10^-4^**  AHSG; ANKH; ARSE; BMP3; BMPR1B; COL10A1; DMP1; DSPP; EBP; ETS2; EVC; FGFR3; GDF10; IBSP; MEPE; MSX1; PITX1; SHOX; SHOX2; SPP1; TUFT1; UFD1L |
| **14) Alcohol dehydrogenase activity; ; Molecular function; 10^-3^**  ADH1A; ADH1C; ADH4; ADH5; ADH6; ADH7; FASN; RTN4IP1 |
| **15) Metal ion homeostasis; Biological Process; 10^-3^**  CCL1; CCL11; CCL13; CCL2; CCL7; CLDN16; MT1A; MT1B; MT1F; MT1G; MT1H; MT1X; MT2A; MT3 |
| **16) MAP kinase activity; Molecular function; 2.10^-3^**  MAPK1; MAPK10; MAPK11; MAPK12; MAPK3; MAPK8 |
| **17) Antiviral response protein activity; Molecular function; 2.10^-3^**  IFNA10; IFNA17; IFNA21; IFNA4; IFNB1; IFNW1; MX1; MX2; RNF4 |
| **18) Development; Biological Process; 2.10^-3^**  ACR; AHSG; AKAP4; ANGPT2; ANKH; ANKRD7; APBA2; ARSE; ARVCF; ASF1A; ATOH1; ATP10A; ATRN; BICC1; BIRC7; BMP15; BMP3; BMPR1B; BTD; C15ORF2; C4ORF6; CCL2; CD164; CHRNA4; CHST8; CLN8; CLTCL1; COL10A1; COL6A3; CRMP1; CRYAA; CSF2RA; CTNND2; CYP46A1; DGCR14; DGCR2; DGCR6; DKK1; DMP1; DMRT1; DMRT2; DMRT3; DOC2A; DRD1; DSCAM; DSP; DSPP; EBP; ELN; ERG; ETS2; EVC; EVL; FABP7; FAT; FGF12; FGF5; FGFR3; FLG; FOXC2; FOXF1; FOXL1; FOXP2; FSCN2; FZD10; FZD6; FZD9; GATA1; GBX2; GDF10; GDF2; GNRH2; GPC1; GTF2IRD1; HAND2; HDAC4; HDAC6; HEY2; HIRA; HSPA2; HYAL4; IBSP; IFRD1; IL11; IL3RA; INSL4; IRX4; KCNQ2; KIAA0776; LAMA2; LETM1; LIMK1; MAEA; MAPK12; MBP; MEOX2; MEPE; MET; MITF; MOV10L1; MSI1; MSX1; MT3; MYH13; MYH8; MYL5; MYOM2; NAGLU; NCOA4; NDN; NEO1; NPY1R; NR2E1; NR4A2; OAZ3; OCA2; PAX4; PCP4; PDCD1; PHYH; PITX1; PIWIL1; PKD2; PLXNB2; PMP22; PPP2CA; PTPRZ1; RET; RFNG; ROD1; RTN4R; SEMA3A; SEMA3D; SEMA5A; SEMA7A; SGCG; SHOX; SHOX2; SIRT4; SIRT7; SLIT2; SMO; SNCA; SPAM1; SPON2; SPP1; SRD5A1; STMN3; T; TBX1; TBX6; TCF15; TLR3; TNRC4; TUFT1; TYRP1; UBE3A; UFD1L; UGCG; UNC5C; WAS; WFS1; WHSC1; WHSC2; WNT16; WNT2; YAF2; ZNF256; ZNF74 |
| **19) Cytokine activity; Molecular function; 3.10^-3^**  BMP15; BMP3; CCL1; CCL11; CCL13; CCL2; CCL7; CCL8; FAM3B; FAM3C; GDF10; GDF2; IFNA10; IFNA13; IFNA14; IFNA16; IFNA17; IFNA2; IFNA21; IFNA4; IFNA5; IFNA6; IFNA8; IFNB1; IFNW1; IL11; PDGFA; SPP1 |
| **20) Cation homeostasis; Biological Process; 4.10^-3^**  CCL1; CCL11; CCL13; CCL2; CCL7; CLDN16; MT1A; MT1B; MT1F; MT1G; MT1H; MT1X; MT2A; MT3; SLC9A3 |
