# Supplementary material for: Structural, Genetic, and Functional Signatures of Disordered Neuro-Immunological Development in Autism Spectrum Disorder
Source: PLoS One. 2012 Dec 4;7(12):e48835. doi: 10.1371/journal.pone.0048835 (PMC3514226; doi:10.1371/journal.pone.0048835)
Supplement: Table S2 — Immune function gene sets from the copy number variation (CNV) regions of autistic individuals. (DOCX) [file pone.0048835.s002.docx]

**Table S2**. Immune function gene sets from the copy number variation (CNV) regions of autistic individuals.

| **Rank 1**  **iCNV-a**  **p value: 9.10^-8^** | **Rank 2**  **iCNV-b**  **p value: 8.10^-6^** | **Rank 10**  **iCNV-c**  **p value: 4.10^-4^** | **Rank 17**  **iCNV-d**  **p value: 2.10^-3^** | **Rank 19**  **iCNV-e**  **p value: 3.10^-3^** |
| --- | --- | --- | --- | --- |
| IFNA10 | IFNA10 | CCL11 | IFNA10 | BMP15 |
| IFNA13 | IFNA13 | CCL8 | IFNA17 | CCL1 |
| IFNA16 | IFNA14 | IFNA10 | IFNA21 | CCL11 |
| IFNA17 | IFNA16 | IFNA17 | IFNA4 | CCL13 |
| IFNA2 | IFNA17 | IFNA21 | IFNB1 | CCL2 |
| IFNA21 | IFNA2 | IFNA4 | IFNW1 | CCL7 |
| IFNA4 | IFNA21 | IFNA6 | MX1 | CCL8 |
| IFNB1 | IFNA4 | IFNB1 | MX2 | FAM3C |
| IFNW1 | IFNA5 | IFNW1 | RNF4 | IFNA10 |
|  | IFNA6 |  |  | IFNA13 |
|  | IFNA8 |  |  | IFNA14 |
|  | IFNB1 |  |  | IFNA16 |
|  | IFNW1 |  |  | IFNA17 |
|  | IL11 |  |  | IFNA2 |
|  |  |  |  | IFNA21 |
