# Supplementary material for: Structural, Genetic, and Functional Signatures of Disordered Neuro-Immunological Development in Autism Spectrum Disorder
Source: PLoS One. 2012 Dec 4;7(12):e48835. doi: 10.1371/journal.pone.0048835 (PMC3514226; doi:10.1371/journal.pone.0048835)
Supplement: Table S3 — Results of LoGS analysis using only genes that were both within 50% recombination distance of the autism loci AND overlapped with CNV's. V = enrichment score. (DOC) [file pone.0048835.s003.doc]

**Table S3.** Results of LoGS analysis using only genes that were both within 50% recombination distance of the autism loci AND overlapped with CNV’s. V = enrichment score.

| Gene set | V | # genes in gene set |
| --- | --- | --- |
| Cytokine activity (**iCNV-e**) | 50 | 20 |
| Hematopoietin/IFN-class cytokine receptor binding (**iCNV-b**) | 41 | 12 |
| Response to virus (**iCNV-c**) | 34 | 8 |
| Interferon-alpha/beta receptor binding (**iCNV-a**) | 34 | 8 |
| Antiviral response protein activity (**iCNV-d**) | 27 | 5 |
| c7 (cell adhesion, ECM) | 26 | 6 |
| c30 (regulation of cell proliferation) | 22 | 4 |
| c29 (perception of light) | 17 | 4 |
| c3 (ribonucleoprotein complex, apoptosis) | 17 | 3 |
| c34 (hydrolase activity, neurogenesis) | 14 | 9 |
