# Supplementary material for: Structural, Genetic, and Functional Signatures of Disordered Neuro-Immunological Development in Autism Spectrum Disorder
Source: PLoS One. 2012 Dec 4;7(12):e48835. doi: 10.1371/journal.pone.0048835 (PMC3514226; doi:10.1371/journal.pone.0048835)
Supplement: Table S4 — Genes in the c6 and c34 gene sets under the LoGS analysis. (DOC) [file pone.0048835.s004.doc]

**Table S4.** Genes in the c6 and c34 gene sets under the LoGS analysis.

| **LoGS over autism loci for the c6 gene set** | | |
| --- | --- | --- |
| Gsn | krt17 | krt38 |
| capn1 | clec3b | krt76 |
| Hdgf | pkp1 | krt2 |
| anxa1 | tuft1 | krt13 |
| Jup | krt1 | dpt |
| flot2 | slc6a6 | ndel1 |
| krt5 | il6r | sf1 |
| Fibp | Roar | krt33a |
| tacstd2 | myl6 | ccnd1 |
| cyp51a1 | c9orf3 | glod4 |
| crabp2 | krt4 | hsd17b7 |
| per1 | krt81 | klf4 |
| s100a4 | lefty2 | krt86 |
| Lmna | lefty1 | krt35 |
| rbms1 | sim2 | klf5 |
| blzf1 | krt31 | lamb3 |
| Rarg | chad | krt14 |
| f3 | krt10 | krt16 |
| Acpp | cdkn2a | rhod |
| rps6ka4 | krt32 | myoc |
| itgb4 | myog | ptprj |
| sprr1b | alox12b | tmbim1 |
| ptgs1 | map2k3 | qars |
| dpp3 | krt83 | crct1 |
| tmem45a | aloxe3 |  |

| **LoGS over autism loci**  **for the c34 gene set** | | |
| --- | --- | --- |
| c11orf58 | chst2 | sept4 |
| App | mapt | scn1a |
| prkar1a | scg2 | scn3a |
| ap2m1 | cdk5 | mllt11 |
| cd81 | atp1b2 | cntn1 |
| pea15 | gap43 | b3galnt1 |
| map4 | pftk1 | ptgds |
| ahcyl1 | tgfbr3 | serpine2 |
| dnaja1 | cadps | rab6ip1 |
| eif4a2 | pfn2 | tuba1b |
| rab14 | atf2 | mapk8ip1 |
| txndc14 | ptprd | rnd2 |
| cstb | sh3gl2 | atp6v0e2 |
| flot2 | wnt10b | sox2 |
| snrpn | rgs7 | oaz3 |
| snurf | bdnf | rap2b |
| ube2n | smarca2 | rap2a |
| ubl3 | accn1 | nfasc |
| dvl3 | cldn11 | wdr6 |
| mycbp2 | hsph1 | copg |
| atp6v1a | gpr37l1 | pcnp |
| stxbp1 | pin1l | npdc1 |
| polr2g | mobp | pfdn2 |
| gtf2h1 | dpp6 | nlrp1 |
| lpgat1 | casc3 | aph1a |
| prkacb | adprh | ptbp2 |
| ssx2ip | ttc3 | rrp1 |
| kif5c | wbp2 | ca14 |
| c11orf49 | tuba1a | gdap2 |
| map2k4 | syt11 | rtn3 |
| kifap3 | plekhb1 | zmat3 |
| gfap | gnai1 | tac3 |
| wbp4 | cd200 |  |
| tnk2 | mrpl9 |  |
| rab21 | cbx5 |  |
| scg5 | atp2c1 |  |
| tubg2 | abca2 |  |
