# Supplementary material for: Structural, Genetic, and Functional Signatures of Disordered Neuro-Immunological Development in Autism Spectrum Disorder
Source: PLoS One. 2012 Dec 4;7(12):e48835. doi: 10.1371/journal.pone.0048835 (PMC3514226; doi:10.1371/journal.pone.0048835)
Supplement: Table S5 — Top Enrichment themes of the c6 and c34 gene sets using EASE. (DOCX) [file pone.0048835.s005.docx]

**Table S5.** Top Enrichment themes of the c6 and c34 gene sets using EASE.

|  | **LoGS over autism loci for the c6 gene set** |
| --- | --- |
| top 3 BP | epidermal differentiation |
|  | ectoderm development |
|  | histogenesis |
| top 3 MF | structural molecule activity |
|  | structural constituent of cytoplasm |
|  | structural constituent of epidermis |
| top 3 CL | intermediate filament |
|  | intermediate filament cytoskeleton |
|  | cytoskeleton |

|  | **LoGS over autism loci for the c34 gene set** |
| --- | --- |
| top 3 BP | neurogenesis |
|  | obsolete biological process |
|  | cellular process |
| top 3 MF | hydrolase activity, acting  on acid anhydrides |
|  | purine nucleotide binding |
|  | nucleotide binding |
| top 3 CL | microtubule associated complex |
|  | microtubule cytoskeleton |
|  | plasma membrane |
