# Supplementary material for: Structural, Genetic, and Functional Signatures of Disordered Neuro-Immunological Development in Autism Spectrum Disorder
Source: PLoS One. 2012 Dec 4;7(12):e48835. doi: 10.1371/journal.pone.0048835 (PMC3514226; doi:10.1371/journal.pone.0048835)
Supplement: Table S6 — LoGS was rerun with different sized windows (forty percent, thirty percent, twenty percent, ten percent, and 5 percent recombination units). V = enrichment score. (DOCX) [file pone.0048835.s006.docx]

**Table S6.** LoGS was rerun with different sized windows (forty percent, thirty percent, twenty percent, ten percent, and 5 percent recombination units). V = enrichment score.

| Rank of 188 gene sets | Forty percent | V | # genes |
| --- | --- | --- | --- |
| 1 | **Cytokine activity (iCNV-e)** | 250 | 20 |
| 2 | **Hematopoietin/IFN-class cytokine receptor binding (iCNV-b)** | 207 | 12 |
| 3 | **Response to virus (iCNV-c)** | 169 | 8 |
| 4 | **Interferon-alpha/beta receptor binding (iCNV-a)** | 169 | 8 |
| 5 | MAP00260_Glycine_serine_and_threonine_metabolism | 124 | 10 |
| 6 | c6 (epidermal differentiation, ectoderm development) | 115 | 70 |
| 7 | c1 (cellular process, cell proliferation) | 113 | 47 |
| 8 | MAP00960_Alkaloid_biosynthesis_II | 111 | 3 |
| 9 | c34 (hydrolase activity, neurogenesis) | 104 | 98 |
| 10 | MAP00531_Glycosaminoglycan_degradation | 102 | 4 |
| 14 | **Antiviral response protein activity (iCNV-d)** | 99 | 7 |

| Rank of 178 gene sets | Thirty percent | V | # genes |
| --- | --- | --- | --- |
| 1 | Cytokine activity **(iCNV-e)** | 216 | 20 |
| 2 | **Hematopoietin/IFN-class cytokine receptor binding (iCNV-b)** | 180 | 12 |
| 3 | **Response to virus (iCNV-c)** | 147 | 8 |
| 4 | **Interferon-alpha/beta receptor binding (iCNV-a)** | 147 | 8 |
| 5 | **Antiviral response protein activity (iCNV-d)** | 116 | 5 |
| 6 | MAP00531_Glycosaminoglycan_degradation | 111 | 3 |
| 7 | c1 (cellular process, cell proliferation) | 106 | 42 |
| 8 | c10 (glutathione transferase activity, epidermal differentiation) | 101 | 53 |
| 9 | c31 (transcription factor activity, cell communication) | 99 | 77 |
| 10 | MAP00960_Alkaloid_biosynthesis_II | 98 | 3 |
| 23 | c6 (epidermal differentiation, ectoderm development) | 81 | 65 |
| 61 | c34 (hydrolase activity, neurogenesis) | 53 | 93 |

| Rank of 170 gene sets | Twenty percent | V | # genes |
| --- | --- | --- | --- |
| 1 | Cytokine activity **(iCNV-e)** | 171 | 20 |
| 2 | **Hematopoietin/IFN-class cytokine receptor binding (iCNV-b)** | 144 | 12 |
| 3 | **Response to virus (iCNV-c)** | 117 | 8 |
| 4 | **Interferon-alpha/beta receptor binding (iCNV-a)** | 117 | 8 |
| 5 | c10 (glutathione transferase activity, epidermal differentiation) | 102 | 42 |
| 6 | MAP00531_Glycosaminoglycan_degradation | 95 | 3 |
| 7 | **Antiviral response protein activity (iCNV-d)** | 93 | 5 |
| 8 | MAP00380_Tryptophan_metabolism | 87 | 12 |
| 9 | MAP00330_Arginine_and_proline_metabolism | 86 | 8 |
| 10 | MAP00590_Prostaglandin_and_leukotriene_metabolism.txt | 83 | 3 |
| 33 | c6 (epidermal differentiation, ectoderm development) | 61 | 55 |
| 64 | c34 (hydrolase activity, neurogenesis) | 47 | 75 |

| Rank of 147 gene sets | Ten percent | V | # genes |
| --- | --- | --- | --- |
| 1 | Cytokine activity **(iCNV-e)** | 82 | 19 |
| 2 | c1 (cellular process, cell proliferation) | 77 | 24 |
| 3 | c6 (epidermal differentiation (BP), ectoderm development) | 66 | 30 |
| 4 | **Hematopoietin/IFN-class cytokine receptor binding (iCNV-b)** | 65 | 12 |
| 5 | MAP00531_Glycosaminoglycan_degradation.txt | 63 | 3 |
| 6 | c12 (endoplasmic reticulum, cell differentiation) | 63 | 21 |
| 7 | c15 (nucleobase\, nucleoside\, nucleotide and nucleic acid metabolism) | 58 | 21 |
| 8 | MAP00340_Histidine_metabolism | 57 | 5 |
| 9 | c18 (muscle contraction) | 55 | 20 |
| 10 | c20 (mitochondrion, energy derivation by oxidation of organic compounds) | 54 | 26 |
| 11 | **Response to virus (iCNV-c)** | 53 | 8 |
| 12 | **Interferon-alpha/beta receptor binding (iCNV-a)** | 53 | 8 |
| 37 | **Antiviral response protein activity (iCNV-d)** | 42 | 5 |
| 124 | c34 (hydrolase activity, neurogenesis) | 12 | 46 |

| Rank of 123 gene sets | Five percent | V | # genes |
| --- | --- | --- | --- |
| 1 | c29 (perception of light) | 57 | 12 |
| 2 | c6 (epidermal differentiation, ectoderm development) | 56 | 20 |
| 3 | MAP00361_gamma_Hexachlorocyclohexane_degradation | 52 | 3 |
| 4 | c1 (cellular process, cell proliferation) | 44 | 18 |
| 5 | c12 (endoplasmic reticulum, cell differentiation) | 44 | 15 |
| 6 | c25 (TF activity, nucleobase, nucleoside, nucleotide and nucleic acid metabolism) | 42 | 4 |
| 7 | c33 (proteasome complex , synaptic transmission) | 41 | 14 |
| 8 | MAP00071_Fatty_acid_metabolism | 41 | 4 |
| 9 | c0 (mitotic cell cycle) | 41 | 16 |
| 10 | c9 (receptor binding, regulation of cellular process) | 40 | 6 |
| 59 | **Cytokine activity (iCNV-e)** | 19 | 6 |
| 70 | c34 (hydrolase activity, neurogenesis) | 16 | 26 |
| 85 | **Response to virus (iCNV-c)** | 13 | 2 |
| *Not found* | ***Interferon-alpha/beta receptor binding (iCNV-a)*** |  |  |
| *Not found* | ***Hematopoietin/IFN-class cytokine receptor binding (iCNV-b)*** |  |  |
| *Not found* | ***Antiviral response protein activity (iCNV-d)*** |  |  |
