# Supplementary material for: Structural, Genetic, and Functional Signatures of Disordered Neuro-Immunological Development in Autism Spectrum Disorder
Source: PLoS One. 2012 Dec 4;7(12):e48835. doi: 10.1371/journal.pone.0048835 (PMC3514226; doi:10.1371/journal.pone.0048835)
Supplement: Table S7 — LoGS without LOD: Except for two gene sets in the lower part of the top 20 ranking all the other gene sets are consistent across the LoGS which use the LOD score and the LoGS without the use of the LOD score. V = enrichment score. (DOCX) [file pone.0048835.s007.docx]

**Table S7.** LoGS without LOD: Except for two gene sets in the lower part of the top 20 ranking all the other gene sets are consistent across the LoGS which use the LOD score and the LoGS without the use of the LOD score. V= enrichment score.

| **Rank**  **(w/o LOD)** | **Gene Set** | **V** | **Rank**  **w/ LOD** | **V**  **w/ LOD** |
| --- | --- | --- | --- | --- |
| 1 | **Cytokine activity (iCNV-e)** | 272 | 1 | 255 |
| 2 | **Hematopoietin/IFN-class cytokine receptor binding (iCNV-b)** | 225 | 2 | 212 |
| 3 | **Response to virus (iCNV-c)** | 184 | 3 | 174 |
| 4 | **Interferon-alpha/beta receptor binding (iCNV-a)** | 184 | 4 | 173 |
| 5 | c6 (epidermal differentiation, ectoderm development) | 133 | 5 | 168 |
| 6 | MAP00960_Alkaloid_biosynthesis_II | 119 | 7 | 119 |
| 7 | c33 (proteasome complex, synaptic transmission) | 119 | 12 | 106 |
| 8 | c34 (hydrolase activity, neurogenesis) | 117 | 6 | 126 |
| 9 | c28 (signal transducer activity, lactose metabolism) | 111 | 14 | 102 |
| 10 | OXPHOS_HG-U133A_probes | 110 | 8 | 119 |
| 11 | MAP00531_Glycosaminoglycan_degradation | 109 | 11 | 108 |
| 12 | **Antiviral response protein activity (iCNV-d)** | 109 | 17 | 100 |
| 13 | MAP00193_ATP_synthesis | 109 | 15 | 101 |
| 14 | MAP03070_Type_III_secretion_system | 109 | 16 | 101 |
| 15 | c1 (cellular process, cell proliferation) | 108 | 9 | 118 |
| 16 | c10 (glutathione transferase activity, epidermal differentiation) | 107 | 10 | 116 |
| 17 | c31 (transcription factor activity, cell communication) | 98 | 18 | 100 |
| 18 | c20 (mitochondrion, energy derivation by oxidation of organic compounds) | 97 | 28 | 89 |
| 19 | MAP00680_Methane_metabolism | 96 | 13 | 103 |
| 20 | fibroblast_serum_response_coag_hemostasis_gsym | 96 | 24 | 92 |
