# Supplementary material for: Structural, Genetic, and Functional Signatures of Disordered Neuro-Immunological Development in Autism Spectrum Disorder
Source: PLoS One. 2012 Dec 4;7(12):e48835. doi: 10.1371/journal.pone.0048835 (PMC3514226; doi:10.1371/journal.pone.0048835)
Supplement: Table S8 — LoGS data input. (DOCX) [file pone.0048835.s008.docx]

**Table S8**. LoGS data input.

|  | Location | Marker | LOD score | Reference | Year |
| --- | --- | --- | --- | --- | --- |
| 1 | 1p21.1 | D1S1631 | 3.44 | Risch | 1999 |
| 2 | 1q23.3 | D1S484 | 3.58 | Ylisaukko-oja | 2004 |
| 3 | 1q42.2 | D1S1656 | 3.06 | Buxbaum | 2004 |
| 4 | 2q31.1 | D2S2188 | 4.8 | International | 2001 |
| 5 | 2q31.1 | D2S335 | 3.32 | Buxbaum | 2001 |
| 6 | 3p24.1 | D3S2432 | 3.32 | Ylisaukko-oja | 2004 |
| 7 | 3q22.1 | D3S3045 | 3.1 | Alarcon | 2005 |
| 8 | 3q26.1 | D3S1763 | 3.1 | Alarcon | 2005 |
| 9 | 3q26.32 | D3S3715,D3S3037 | 4.81 | Auranen | 2002 |
| 10 | 7q22.1 | D7S477 | 3.55 | IMGSAC | 2001 |
| 11 | 7q32.1 | D7S530 | 3.55 | IMGSAC | 1998 |
| 12 | 7q34 | D7S684 | 3.55 | IMGSAC | 1998 |
| 13 | 7q36.1 | D7S483 | 3.7 | Molloy | 2005 |
| 14 | 7q36.3 | D7S2462 | 3.66 | Auranen | 2002 |
| 15 | 9p22.2 | D9S157 | 3.11 | IMGSAC | 2001 |
| 16 | 9q34.3 | D9S1826 | 3.59 | IMGSAC | 2001 |
| 17 | 12q14.2 | rs1445442 | 4.51 | Ma | 2007 |
| 18 | 13q22.1 | D13S800 | 3 | Barrett | 1999 |
| 19 | 15q12 | GABRB3 | 4.71 | Shao | 2003 |
| 20 | 17q11.2 | D17S1294 | 4.3 | Stone | 2004 |
| 21 | 17q11.2 | D17S1294 | 8 | Sutcliffe | 2005 |
| 22 | 17q11.2 | D17S1800 | 8 | Sutcliffe | 2005 |
| 23 | 17q11.2 | D17S798 | 4.3 | Stone | 2004 |
| 24 | 17q21.2 | D17S1299 | 4.6 | Cantor | 2005 |
| 25 | 17q21.32 | D17S2180 | 4.1 | Cantor | 2005 |
| 26 | 21q21.1 | D21S1437 | 3.4 | Molloy | 2005 |
| 27 | 11p13 | rs2421826 | 3.57 | Autism Genome Consortium | 2007 |
| 28 | 11p13 | rs1358054 | 3.9 | Autism Genome Consortium | 2007 |
| 29 | 9p24.1 | rs722628 | 3.59 | Autism Genome Consortium | 2007 |
| 30 | 9q33.3 | rs536861 | 3.3 | Autism Genome Consortium | 2007 |
